# Suppress Austenite Grain Coarsening by Nb Alloying in High–Temperature–Pseudo–Carburized Bearing Steel

**DOI:** 10.3390/ma17122962

**Published:** 2024-06-17

**Authors:** Xueliang An, Wenquan Cao, Xiaodan Zhang, Jinku Yu

**Affiliations:** 1 State Key Laboratory of Metastable Materials Science and Technology, Yanshan University, Qinhuangdao 066004, China; anxueliang@stumail.ysu.edu.cn (X.A.); yujinku@ysu.edu.cn (J.Y.); 2 Central Iron and Steel Research Institute (CISRI), Beijing 100081, China; 3 Section of Manufacturing Engineering, Department of Civil and Mechanical Engineering, Technical University of Denmark, 2800 Kongens Lyngby, Denmark

**Keywords:** Nb alloying, precipitate, refined grain, growth dynamics

## Abstract

The effect of Nb alloying on the suppression of austenite grain coarsening behavior during pseudo–carburizing is investigated in high–temperature–carburized SAE4320 bearing steel. To explore the role of the Nb element in the pseudo–carburizing process, the morphology, composition, size, and distribution of NbC precipitates were analyzed. The results show that the fine austenite grain observed in Nb micro–alloyed steel is caused by the pinning effect of NbC precipitates, which hinders the coarsening of austenite grains and changes the growth dynamics of austenite grains. After the SAE4320 carburized bearing steel with the addition of 0.45 wt.% Nb element is kept at 1150 °C for 4 h, the PAG size is still below 20 μm, which indicates the Nb element has obvious advantages in limiting PAG growth at high temperatures and shows great potential for the development of high–temperature carburized bearing steel.

## 1. Introduction

Bearings work as key precision components in a broad scope of machinery and are vital machine elements [1,2,3]. SAE4320 steel is widely applied in bearings and gears in the case–hardened state, such as carburizing and nitriding, as the combination of soft matrix and hard surface introduces superior wear resistance, excellent fatigue properties, and outstanding toughness [4,5]. The traditional gas carburizing temperature of SAE4320 bearing steel is around 930 °C, and the carburizing time usually takes a few hours to a few days according to the required thickness of the carburizing layer. The emergence of high–temperature carburizing above 930 °C greatly reduces the carburizing time and improves the efficiency of carburizing [6]. The carburizing process is mainly affected by the diffusion process and diffusion coefficient. When the carburizing temperature is increased from 950 °C to 1050 °C, the carburizing time can be shortened to about 1/3 of the original [7]. However, high–temperature carburizing is usually accompanied by the additional growth of austenite grains, resulting in significant grain coarsening in most of the current industrial bearing steels [8]. The occurrence of such additional grown grains is usually considered to be caused by the lack of pinning from the precipitates [9,10]. Grain size is one key microstructural parameter for mechanical properties of bearing steels, such as strength and fatigue limit [11], similar to the single–phase [12,13,14] and multiple–phases [15,16,17] polycrystalline metals and alloys. Grain coarsening introduced by high–temperature carburizing will inevitably lead to a reduction in properties and performance, so it is necessary to refine the grains. Microalloying is an effective way to refine grains and inhibit grain growth. Usually, the distribution of precipitated phases is optimized by adding strong precipitate–forming alloy elements, such as Nb and V [18], to inhibit the coarsening of prior austenite grains (PAGs) during carburizing [11,19]. Previous studies reported that the addition of microalloying elements would bring about fine precipitates, which would pin austenite grains boundaries during the process of austenite grains growth and inhibit their growth process [20]. It has also been suggested that Nb atoms, which are solidly dissolved, can drag the grain boundary [21,22]. At present, the addition of Nb element in bearing steels is normally less than 0.1%, and there is a lack of systematic research, especially on the mechanisms of inhibiting grain coarsening above 1000 °C.

Therefore, this paper simulates the grain coarsening phenomenon that may occur in the high–temperature carburizing process of bearing steel and explores the influence of the pseudo–carburizing process at different temperatures on grain size. The effect of Nb content on grain size after holding at different temperatures was investigated by adding different Nb contents (0.05%, 0.1%, 0.2%, 0.5%). The grain growth process was observed in real–time through a high–temperature metallographic test, and the changes in precipitated phases relative to grain boundaries during grain growth were observed. The qualitative, quantitative, and size distribution of the precipitated phase were analyzed. The effect of different Nb contents on the evolution of precipitated phase content and size is further clarified, as well as the coarsening dynamics of PAG during the pseudo–carburizing of Nb micro–alloyed steels.

## 2. Materials and Methods

The four experimental steels were refined in a vacuum induction furnace and then cast into ingots. The ingot was homogenized so that the micro–alloyed elements were uniformly dissolved in the steel. The ingot was then reheated to 1200 °C for 2 h and forged to a bar diameter of 15 mm. The chemical compositions of four experimental steels are shown in Table 1. A sample with a diameter of 15 mm and a thickness of 7 mm was obtained from the forging.

The samples were pseudo–carburized (austenitizing in a non–carburizing atmosphere) at 900, 950, 1000, 1050, 1100, and 1150, and then oil quenched. The pseudo carburization time was 0.5, 1, 2, and 4 h, respectively. PAG boundary corrosion of pseudo–carburized samples was carried out in a supersaturating solution of picric acid (add 5 g picric acid crystals into 100 mL water to prepare a supersaturated solution of picric acid). The size of PAG was analyzed by an optical microscope (OM, Olympus–GC51, Olympus, Tokyo, Japan).

The microstructure was observed by scanning electron microscopy (SEM, FEI–Quanta 650FEG, Quanta, Houston, TX, USA). Electron backscatter diffraction (EBSD, JEOL–7200Fm JEOL, Tokyo, Japan) was applied to analyze the phases. Transmission electron microscopy (TEM, Thermo Scientific Talos F200X, Thermo Scientific, Waltham, MA, USA) was applied to investigate the fine precipitated phase, and its chemical composition was qualitatively analyzed by an energy dispersive spectrometer (EDS, Oxford X–Max, Oxford Instruments, Abingdon, UK). The growth of austenite grains was observed in situ by laser scanning confocal microscopy (LSCM, VL2000DX–SVF17SP, LSCM, Paris, France). For in situ observations, the cylindrical sample has a diameter of 7.3 mm and a height of 3 mm, with top and bottom planes mechanically polished. The specimens were heated to 1200 °C with a heating rate of 500 °C/min, isothermally held for 1 h in situ observation, and then cooled down to room temperature at the same rate as heating.

## 3. Results

### 3.1. Effect of Nb Alloying on PAG Size

**Figure 1:**
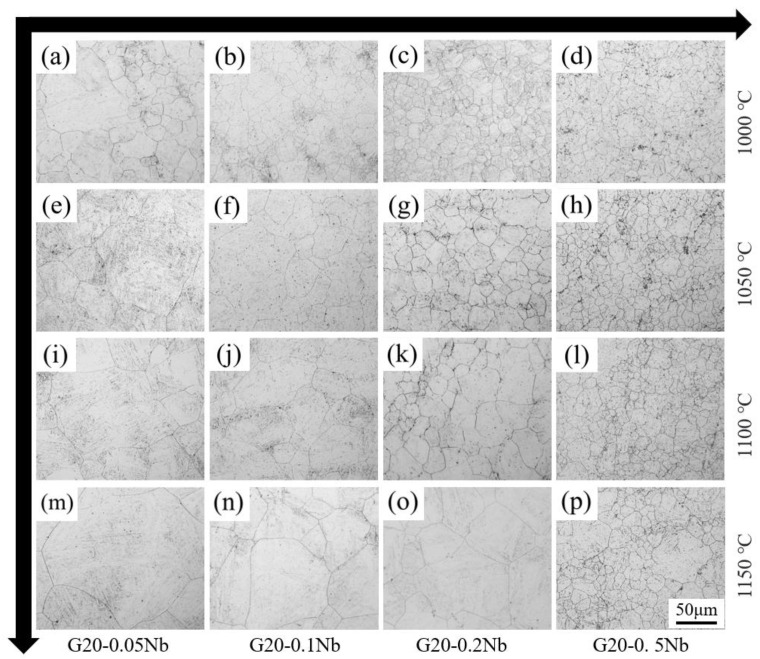
Microstructure of PAG boundary of different steels at different temperatures for 1 h. (**a**) G20–0.05Nb–1000 °C, (**b**) G20–0.1Nb–1000 °C, (**c**) G20–0.2Nb–1000 °C, (**d**) G20–0.5Nb–1000 °C, (**e**) G20–0.05Nb–1050 °C, (**f**) G20–0.1Nb–1050 °C, (**g**) G20–0.2Nb–1050 °C, (**h**) G20–0.5Nb–1050 °C, (**i**) G20–0.05Nb–1100 °C, (**j**) G20–0.1Nb–1100 °C, (**k**) G20–0.2Nb–1100 °C, (**l**) G20–0.5Nb–1100 °C, (**m**) G20–0.05Nb–1150 °C, (**n**) G20–0.1Nb–1150 °C, (**o**) G20–0.2Nb–1150 °C, (**p**) G20–0.5Nb–1150 °C.

To investigate the effect of Nb alloying contents on the PAG size, the samples with different Nb content annealed at four different temperatures for 1 h were grain boundary corroded with the results shown in Figure 1. It can be seen that with the increase in Nb content, the PAG size becomes finer, and the grain size of G20–0.5Nb steel is the smallest at all temperatures. For specimens with the same Nb content, the PAG size becomes coarser with the temperature increase, and the PAG size of sample G20–0.05Nb increases the most.

**Figure 2:**
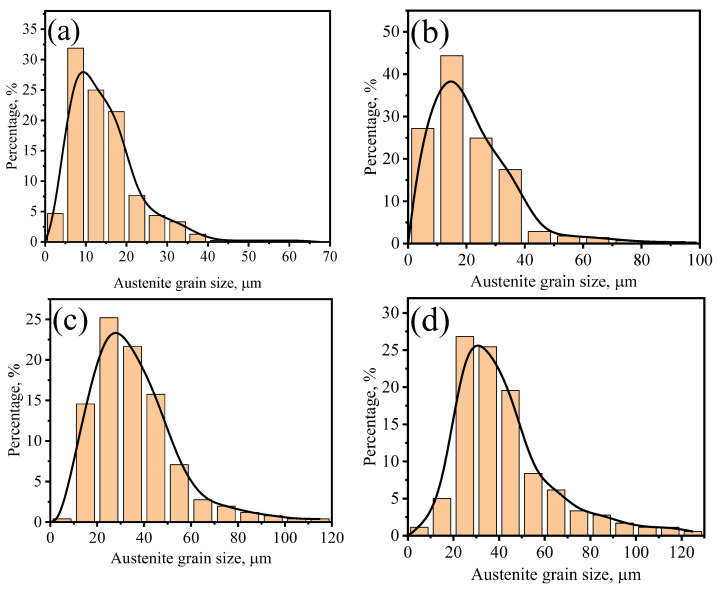
The PAGs distribution of steel G20–0.2Nb specimens kept at different temperatures for 1 h. (**a**) 1000 °C, (**b**) 1050 °C, (**c**) 1100 °C, and (**d**) 1150 °C.

**Figure 3:**
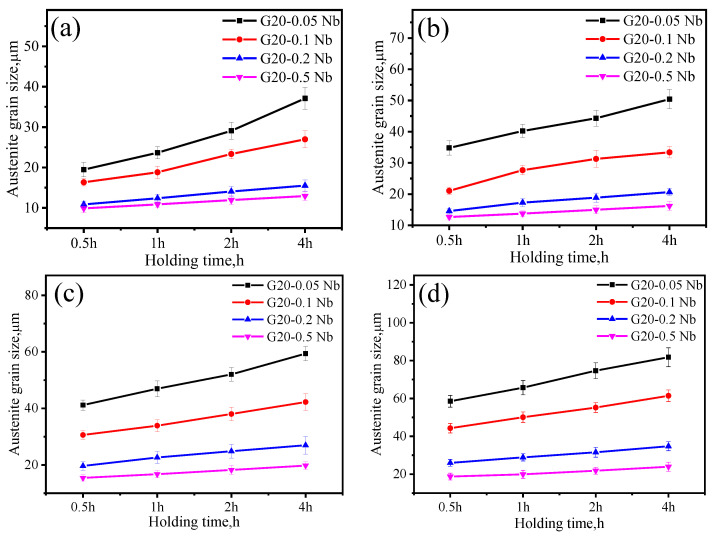
Changes in PAG size of four steels versus the holding time at different temperatures: (**a**) 1000 °C, (**b**) 1050 °C, (**c**) 1100 °C, and (**d**) 1150 °C.

To investigate the coarsening dynamics, the distributions and the average value of PAGs in four different steels are compared. Figure 2 shows the PAGs distribution after 1 h holding at four different pseudo–carburizing temperatures for G20–0.2Nb. It is found that the grain size distribution maintains the unimodal shape, although the average PAG increases from 13.38 μm at 1000 °C to 28.86 μm at 1150 °C. The average PAG under all pseudo–carburizing temperatures and holding times were measured and plotted in Figure 3. With the temperature increase, the PAGs of the steel with the highest Nb content show the minimum growth. The steel with the minimum Nb content has the largest PAGs at all conditions, while almost all the steels show a linear PAG growth with the holding time.

### 3.2. The Appearance of Abnormally Large Grains

**Figure 4:**
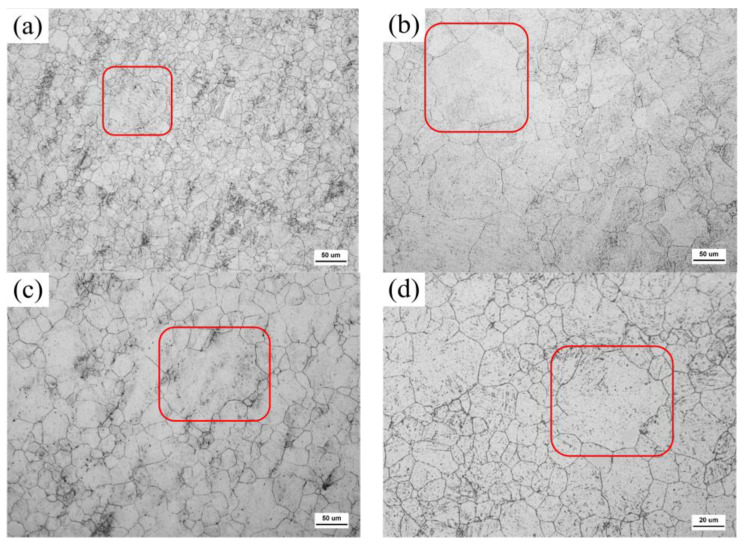
Optical micrographs showing the abnormal large grains in four steels: (**a**) G20–0.05Nb at 1000 °C for 0.5 h, (**b**) G20–0.1Nb at 1050 °C for 1 h, (**c**) G20–0.2Nb at 1100 °C for 2 h, and (**d**) G20–0.5Nb at 1150 °C for 4 h.

Abnormally large grains usually appear at high temperatures. It is interesting that the temperatures and holding times for the appearance of the abnormally large grains increase with the Nb content in the order for G20–0.05Nb to G20–0.5Nb: 1000 °C for 0.5 h, 1050 °C for 1 h, 1100 °C for 2 h, and 1150 °C for 4 h, as evidenced in the microstructures shown in Figure 4.

### 3.3. MC Carbides

**Figure 5:**
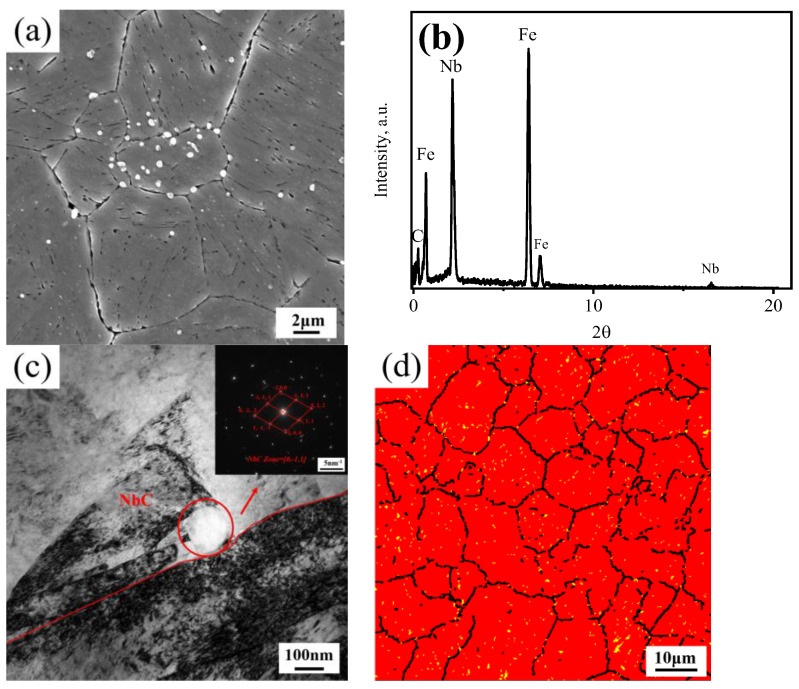
G20–0.5 steel at 1000 °C–1 h: (**a**) the SEM image; (**b**) EDS spectra of the precipitated MC carbides; (**c**) TEM image and selected area diffraction pattern of NbC precipitates at grain boundaries; (**d**) the EBSD micrograph where the red phase is martensite, the yellow is NbC carbides, and the black lines are reconstructed as austenitic grain boundaries with misorientation angle Ɵ > 15°.

To identify the precipitated phases by adding Nb element, scanning electron microscopy and transmission electron microscopy were applied. Figure 5 shows the microstructure of G20–0.5Nb after holding at 1000 °C for 1 h. It can be seen that a large number of precipitated phases are distributed both in the PAGs and at the prior austenite grain boundaries, and the effective pinning of the grain boundary makes the average PAG relatively small, around 11 μm, as shown in Figure 3. The EDS result in Figure 5b and the diffraction pattern in the upper right corner of Figure 5c confirm that the white and round precipitated phases in Figure 5a,c are NbC carbides. The EBSD microstructure characterization of G20–0.5Nb samples annealed at 1000 °C for 1 h was carried out, with the result shown in Figure 5d. It is found that fine NbC carbides exist both in the PAGs and at the prior. 

### 3.4. In Situ Observation of Grain Boundary Unpinning Processes from Carbides

To further understand the effect of alloying elements and carbides on the suppression of austenite grain coarsening, in–situ LSCM with heat treatment up to 1200 °C was applied. The reason for the determination of observation temperature at 1200 °C was the clear observation of austenite grain boundaries from the mechanically–polished surfaces due to the surface diffusion–controlled grain boundary grooving [23]. During the whole heating and cooling processes, although the martensite transformation and the fast, small grain growth below 1200 °C have also been observed, the focus of this part is put on the grain growth and the pinning effect of NbC carbides on the austenite grain boundaries at 1200 °C.

**Figure 6:**
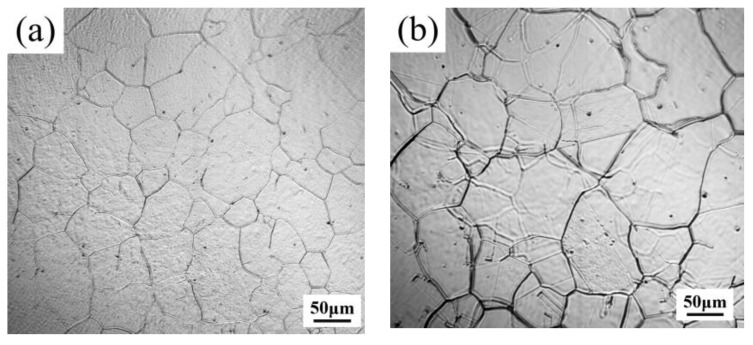
Microstructure screenshots from the LSCM video of the G20–0.05 steel: (**a**) the earliest with austenite grains clearly observed; (**b**) microstructure before cooling.

From the real–time observation of G20–0.05 steel by LSCM, it can be seen that when the grain boundary is fully displayed (microstructure at 411 s in Figure 6a), the grain size has reached a similar grain size as that after 1150 °C pseudo–carburizing for 2 h. The grain boundary morphology with the evolution traces at the end of heating is shown in Figure 6b (microstructure at 3839 s), where the grain size reaches 80 μm, similar to that after pseudo–carburizing at 1150 °C for 4 h.

**Figure 7:**
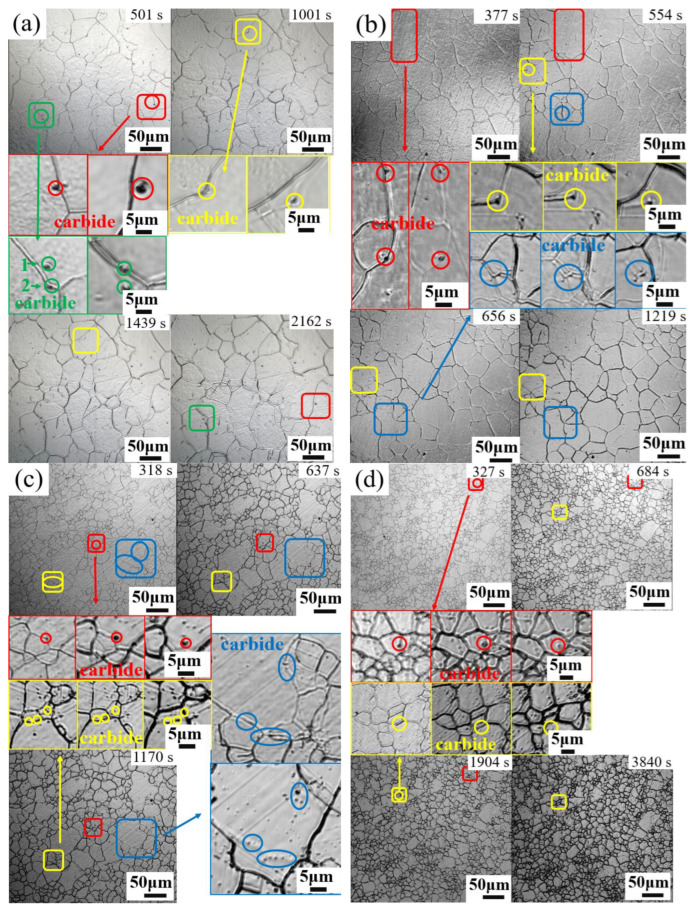
Microstructural snapshots from in –situ LSCM observation at 1200 °C (The carbides of the same color are the states of the same carbides at different holding times): (**a**) G20–0.05Nb; (**b**) G20–0.1Nb; (**c**) G20–0.2Nb; (**d**) G20–0.5Nb.

To show the detailed processes of pinning and depinning of grain boundaries, a series of snapshots were taken from the in –situ LSCM videos of four specimens, as shown in Figure 7. It was found that carbides with red, yellow, and blue frames play a pinning role on the grain boundaries in all four steels, and with the increase in time, the curvature radius of a moving boundary pinned by an NbC carbide decreases sharply; finally, the boundary is depinned.austenite grain boundaries, consistent with the SEM observation.

## 4. Discussion

The above experimental results show that the austenite grain refinement is due to the Nb element addition and that the average PAGS gradually decreases with the increase in Nb content. It is interesting that the abnormal grain growth for different steels appears at different temperatures and holding times. The detailed mechanisms behind this will be discussed in the following sections.

### 4.1. The Appearance and Causes of Abnormally Grown PAGs

Section 3.2 shows the abnormally large PAGs, which appear at 1000 °C, 1050 °C, 1100 °C and 1150 °C in four steels, and from the in situ LSCM observations, the appearance of these large grains can be easily observed. In these steels with Nb micro–alloying, because the Nb element has a larger atomic radius than Fe, it is easy for Nb to segregate at the phase interfaces, resulting in a strong drag effect on the phase interfaces at high temperatures. When the temperature drops below the Nb solution temperature, MC particles precipitated have a strong pinning effect [24,25]. Therefore, in Nb–containing steels, the PAG growth is significantly inhibited, resulting in PAG refinement. When MC carbides are clustered in one area, PAGs are refined, but in the area with fewer MC carbides, the grains are coarse.

The effect of alloying on PAG growth reflects the competition between the normal PAG growth driving forces and the pinning/drag exerted by the precipitated phases/element segregation. In the process of high–temperature holding, precipitates often dissolve or grow up, resulting in partial loosening of PAG boundaries. If some grains are not pinned and others are still pinned, abnormal grain growth begins over a certain temperature/time range [25]. Temperature mainly changes the condition of abnormal grain growth, and the dissolution and growth of precipitates are considered to be the controlling factors. In this study, the casting process, process history, and pseudo–carburizing conditions of all steels were the same except for different Nb alloy contents. The difference in grain coarsening of four steels is caused by the difference in Nb contents and MC precipitates. The present study shows that Nb alloy has great potential to reduce the sensitivity of carburized steel to abnormal grain growth above 1000 °C.

### 4.2. Relationship between Nb Content and Refining Ability

**Figure 8:**
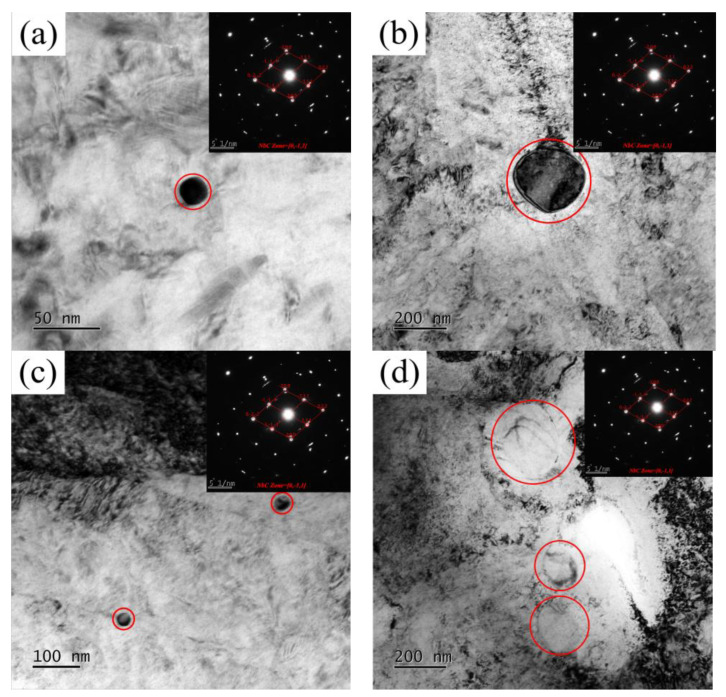
TEM micrographs of the precipitated NbC phases: (**a**) G20–0.05Nb–1000 °C–1 h, (**b**) G20–0.05Nb–1050 °C–1 h, (**c**) G20–0.5Nb–1000 °C–1 h, (**d**) G20–0.5Nb–1150 °C–1 h.

The carbides after pseudo–carburizing were observed by TEM. In Nb–micro–alloyed steels, after being held at 1000 °C, 1050 °C, 1100 °C and 1150 °C, fine and uniform precipitated phase MC was observed, as shown in Figure 8. The small MC precipitates inhibit grain growth through pinning PAG boundaries. With the temperature increase, the solubility and coarsening rate of the precipitates were improved while the stability of the precipitates decreased.

As mentioned above, at the same austenitizing temperature, with the increase in Nb content, the precipitates in alloyed– steels increase, which can pin grain boundaries more effectively. The addition of Mo decreases the growth rate of PAG in Nb–alloyed steels. Previous studies have shown that the addition of Mo has a significant effect on the evolution of precipitate size distribution and PAG refinement in Nb–micro–alloyed steels [26].

It has been [25] proposed that the Mo element can segregate to the precipitate–matrix interface and reduce the precipitate–matrix interface mobility by reducing the surface energy of the precipitate–matrix or the resistance of solute, thus reducing the coarsening rate of the precipitated phase. The Mo element results in a decrease in interfacial energy and maintains a better coherence between the precipitate and the ferrite matrix. A similar hypothesis [27] has been proposed for the incorporation of the Mo element into Nb precipitates. When the Mo element was mixed with the Nb precipitate, the surface energy of the precipitated phase and matrix decreased significantly, and the precipitate coarsened through the mechanism of precipitate reflux [28]. On the other hand, the lower interfacial energy between the precipitate and the matrix reduces the nucleation barrier and the critical nucleation radius. The role of the Mo element in reducing the critical nucleation radius of the MC precipitates at the initial stage of precipitation makes their size relatively small. Therefore, the addition of Nb is conducive to inhibiting PAG coarsening and delaying the occurrence of abnormal grain growth.

TEM observations showed that there was a small amount of MC precipitates in G20–0.05Nb steel when it was held at 1000 °C for 1 h. MC precipitates began to coarsen at 1150 °C, resulting in weakened pinning ability, which is consistent with the abnormal grain growth observed at 1150 °C for pseudo–carburized 1 h Nb–micro–alloyed steel. However, the volume fraction of the MC precipitates in the G20–0.5Nb steel is sufficient to delay the abnormal austenite grain coarsening when it is held at 1000 °C for 1 h. Therefore, the PAG size of G20–0.5Nb steel has little change after pseudo–carburizing at 1000 °C. This is because some MC precipitates dissolved and/or coarsened to a certain extent after being held at 1000 °C for 1 h, but a large part of them did not exceed the critical size. Decreasing the volume fraction of precipitates and increasing the size of precipitates will accelerate the growth of abnormal grains. This situation can be seen when G20–0.5 steel is insulated at 1150 °C for 1 h. This may be related to the rapid coarsening and dissolution of MC precipitates. Although the average PAG size increases to some extent, there are still fine MC precipitates that can effectively pin the grain boundaries, which is also the reason why the average PAG size of G20–0.05 steel is 3 times that of G20–0.5NB steel after being held at 1150 °C for 1 h.

This study shows that the change in the MC precipitates is the main reason for the change in grain size, and the rise of temperature leads to the dissolution and growth of the precipitated phase, consistent with the literature [21].

First of all, the atomic size of Nb is very different from that of Fe, so it has a certain solute atomic drag effect. When a large number of solute atoms such as Nb are reconcentrated on the grain boundaries or subgrant boundaries, the migration of grain boundaries can be limited.

In addition, the essence of austenite grain growth is the migration of grain boundaries, and NbC precipitates can pin austenite grain boundaries and limit the growth of austenite grains. The pinning effect of precipitates can be explained by the driving force of grain growth, *P_G_* [28,29]:

(1) $$P_{G}=\frac{2\sigma }{\rho }$$

where *P_G_* is the driving force of grain growth, MPa; *σ* is the interface energy, J/m^2^; ρ is the radius of curvature, μm.

The pinning force of precipitation relative to grain boundaries can be expressed by the Zener’s model [30]:

(2) $$P_{Z}=\frac{3\sigma F_{V}}{d}$$

where *P_Z_* is the pinning force, MPa; *d* is the precipitate diameter, μm; *F_V_* is the volume fraction of the precipitates.

Equation (2) shows that the smaller the size of the precipitated phase, the larger the volume fraction, and the stronger the pinning force will be. The above Equations (1) and (2) reflect the fact that to guarantee the pinning effect, the number of NbC precipitates must be large enough, and the average size should be small enough. When the grain boundary is pinned by the NbC precipitates, the grain boundary tends to bend to increase the surface energy, as shown in Figure 7, and the migration of the grain boundary is limited. With the increase in time, the curvature radius of a moving boundary pinned by an NbC precipitate decreases sharply and, thereby, increases the grain growth driving force. Only when the driving force *P_G_* is greater than the pinning force *P_Z_* can the boundary become depinned, and the grain boundary can migrate again.

Therefore, NbC precipitates significantly slow down the austenite growth rate and effectively limit the growth of grains. When the pseudo–carburizing temperature and time are high enough or long enough, the area density of dispersed nano NbC precipitates decreases significantly, and the area density of large–size NbC precipitates increases, which weakens the pinning effect of grain boundaries, resulting in austenite grain coarsening.

**Figure 9:**
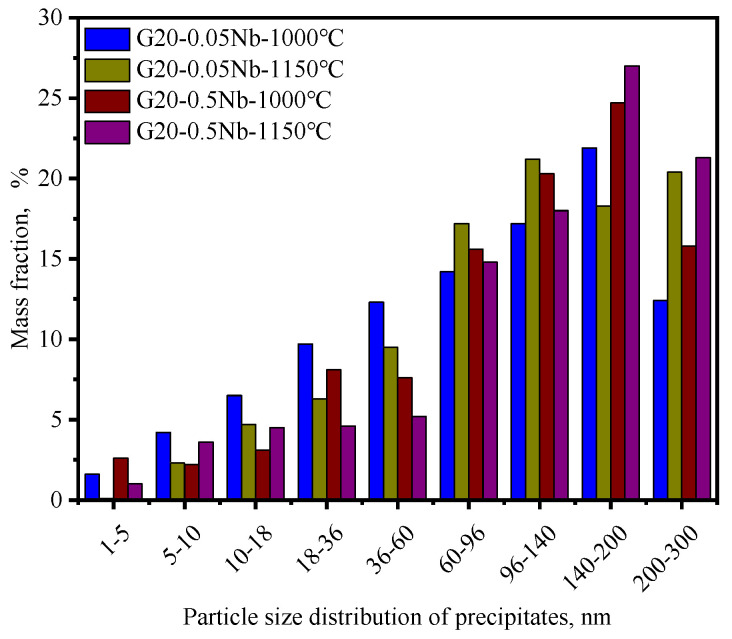
Particle size distribution of the precipitates.

Figure 9 shows the particle size distribution of precipitates. It can be seen from the figure that there is little difference in the particle size distribution of precipitates among the four steels, and the difference in precipitate content leads to a huge difference in the number of precipitates that can provide pinning force in the four steels.

**Figure 10:**
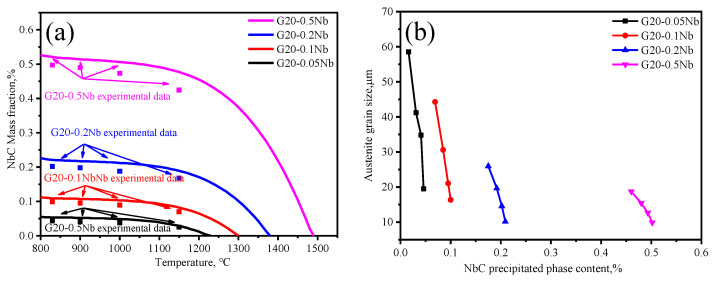
(**a**) The calculated NbC mass fraction compared with the experimental data (**b**) austenite grain size versus NbC mass fraction.

The variation in the precipitate content with temperature obtained through calculation and phase analysis in comparison with experimental data is shown in Figure 10a. It can be seen that with the increase in Nb content, the NbC mass fraction increases. The huge difference in the NbC content results in four different average austenitic grain sizes in four steels: 23.89 μm, 34.74 μm, 61.41 μm, and 81.77 μm.

The precipitated MC carbides have a significant influence on austenite grain growth. Therefore, the theoretical calculation of the solid solution and precipitated phase content of Nb is carried out, and the change law of its solid solution and precipitated phase content is obtained. The equilibrium solid solution content at temperature *T* can be calculated by using the equilibrium solid solubility formula of the unit second phase MX in steel [31]:

(3) $$\left[M\right]{\left[X\right]}^{x}=10^{A-\frac{B}{T}}$$

(4) $$\left[M\right]{\left[X\right]}^{x}=10^{A-\frac{B}{T}}$$

where A and B are constants in the formula of solid solubility of the MX phase in the iron matrix; A_M_ and A_X_ are the atomic weights of elements M and X, respectively. M and N are the test content of the M element and N element in the sample, and [M] and [N] are the solid solution content of the M element and N element, respectively. *T* is the absolute heating temperature (K). In this paper, M is Nb and X is C, so A_M_ is 92.9064 and A_X_ is 12.011.

The solid solution amount of Nb element in the matrix and the volume fraction of NbC precipitated phase can be obtained by Equations (3) and (4):

(5) $$f=M-[M]\cdot \frac{A_{M}+xA_{X}}{A_{M}}$$

The formula for calculating the solid solubility product of NbC selected in this paper is:

(6) $$\mathrm{lg}\left[\mathrm{Nb}\right]\left[C\right]=2.96-\frac{7510}{T}$$

Therefore, after the solid solution content is calculated by Equation (6), the precipitated phase content can be calculated by Equation (5). The change of precipitated phase content with temperature obtained through calculation and simulation is shown in Figure 10a. It can be seen that with the increase in temperature, the content of the precipitated phase will decrease. As can be seen from the figure, the mass fraction of precipitated NbC phases of G20–0.5Nb is about 3 times that of G20–0.2Nb, 5 times that of G20–0.1Nb, and 10 times that of G20–0.05 steel.

There is a difference between the precipitated phase content of G20–0.5Nb steel and the other three steels, which makes the grain of G20–0.5Nb steel significantly refined when compared with others. The average austenite grain sizes of G20–0.05Nb steel, G20–0.1Nb steel, G20–0.2Nb steel, and G20–0.5Nb steel are 23.89 μm, 34.74 μm, 61.41 μm, and 81.77 um, respectively. The G20–0.05Nb sample has nearly 1/3 of the precipitated phase dissolved at 1000 °C, and the dissolution of the precipitates will weaken the pinning effect so that the grain size increases at the corresponding temperature. The results of summarizing the content of the precipitated phase and the grain size are shown in Figure 10b. It can be seen from the diagram that with the increase in Nb content, the grain size becomes finer and finer because there are enough precipitates that can effectively pin the grain boundaries and limit the migration of austenite grain boundaries.

### 4.3. Dynamics of Grain Growth

From the perspective of thermodynamics and dynamics, the growth of PAGs is a heat–activated migration process. The dynamics of grain growth have been thoroughly studied. At present, many models can describe grain growth. The Sellars model has good general applicability, so this paper selects the Sellars model. According to the Sellars model, grain growth behavior follows the following equation [31]:

(7) $$\begin{array}{c} D=k{\left[t\times \exp\left(-\frac{Q}{RT}\right)\right]}^{n} \end{array}$$

where *D* is the average grain size (m), *t* is the holding time (s), *Q* is the activation energy of grain growth (J/mol), *T* is the absolute heating temperature (K), *R* is the gas constant (8.31 J mol^−1^K^−1^), and k and n are the material constants.

When the heating temperature *T* is constant, the logarithm of both sides of Formula (7) is taken at the same time the partial derivative is obtained, and the formula is obtained:

(8) $$n={\left\{\frac{\partial \ln D}{\partial \ln t}\right\}}_{T=\mathrm{const}}$$

**Figure 11:**
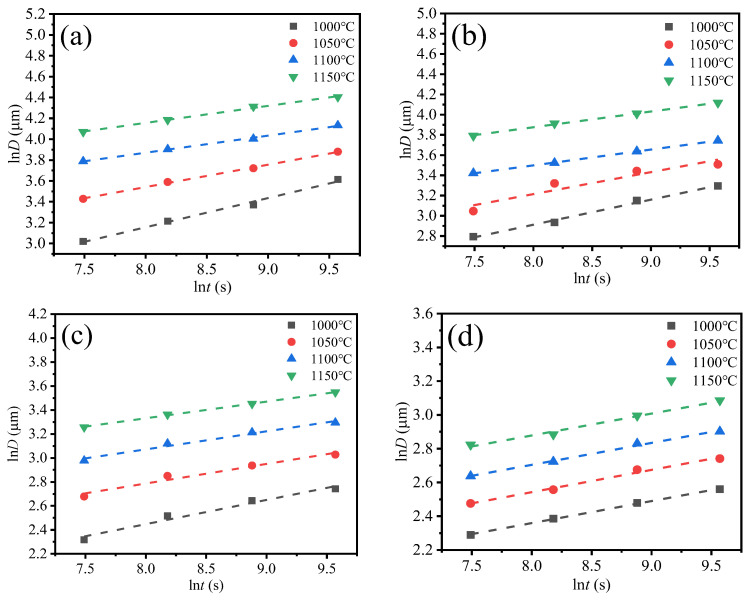
Function diagrams of ln *D* and ln *t* for four steels: (**a**) G20–0.05Nb, (**b**) G20–0.1Nb, (**c**) G20–0.2Nb, (**d**) G20–0.5Nb.

According to Equation (7), Figure 11 can be obtained, and the n value can be calculated. The diagram shows the relationship between ln*D* and ln*t* of four groups of samples.

When the holding time *t* is constant, the logarithm of both sides of Equation (7) is taken at the same time, and the partial derivative is obtained.

(9) $$\begin{array}{c} Q=-\frac{R}{n}{\left\{\frac{\partial \ln D}{\partial \left(\frac{1}{T}\right)}\right\}}_{t=\mathrm{const}} \end{array}$$

**Figure 12:**
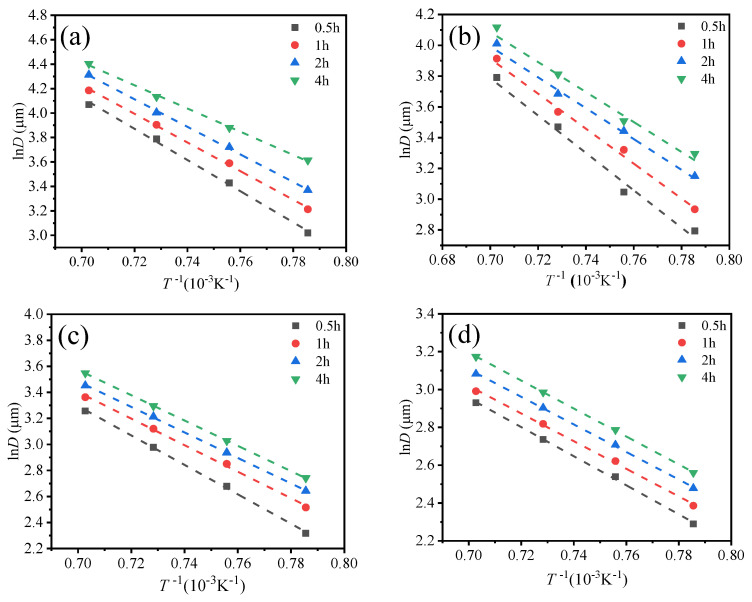
In *D* curve of four kinds of steel with 1000/*T*: (**a**) G20–0.05Nb, (**b**) G20–0.1Nb, (**c**) G20–0.2Nb, (**d**) G20–0.5Nb.

The *Q* value can be obtained according to Equation (9), and Figure 12 shows the relationship between 1000/*T* and ln*D*. The *Q* value of the G20–0.05Nb sample is 467 kJ/mol, the *Q* value of the G20–0.1Nb sample is 495 kJ/mol, the *Q* value of the G20–0.2Nb sample is 546 kJ/mol, and the *Q* value of G20–0.5Nb sample is 609 kJ/mol. The highest *Q* value of the G20–0.5Nb sample is 609 kJ/mol, which further indicates that it has the strongest ability to inhibit grain coarsening. This is also the reason why the G20–0.5Nb sample has the highest temperature at 1150 °C for the appearance of abnormally grown austenite grains.

**Figure 13:**
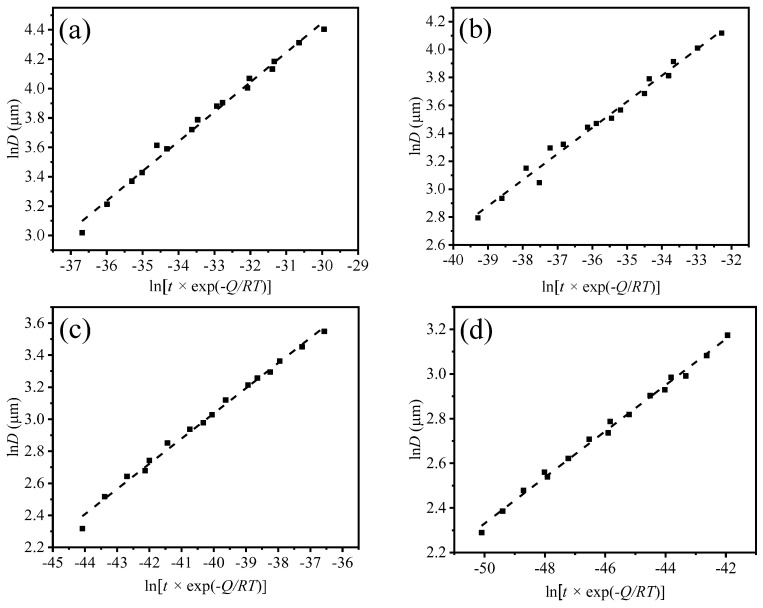
Four steel ln *D* with ln [*t* × exp (−*Q*/*RT*)] change curve (**a**) G20–0.05Nb, (**b**) G20–0.1Nb, (**c**) G20–0.2Nb and Nb (**d**) G20–0.2Nb.

The activation energy *Q* value and parameter n value are brought into the k value, and the change curve of ln *D* with ln[*t* × exp (−*Q*/*RT*)] is drawn, as shown in Figure 13. The fitting results are shown in Table 2, and the linear correlation coefficient after fitting is 0.98–0.99, so it is considered that ln *D* and ln[*t* × exp (−*Q*/*RT*)] conform to the linear relationship. Therefore, alloy additives can significantly change the grain growth mechanism of the matrix steel. The grain coarsening temperature of bearing steel can be increased by adding Nb alloy. The change in *Q* value is due to the presence of precipitates. With the increase in Nb content, the grain coarsening behavior of austenite also has a significant difference.

## 5. Conclusions

Four Nb contents of 0.05 wt.%, 0.1 wt.%, 0.2 wt.%, 0.5 wt.% were added to SAE4320 steel, and the effect of Nb alloying on the suppression of austenite grain coarsening behavior during pseudo–carburizing was investigated. The following conclusions can be drawn.

(1) The PAG is gradually refined with an increase in Nb content. The grain size is refined from 20 μm of the G20–0.05Nb steel to 10 μm of the G20–0.5Nb steel at 1000 °C for 0.5 h.

(2) The austenite grain growth was due to the curvature radius decrease of a moving boundary pinned by an MC precipitate and the gradual growth and dissolution of MC precipitates with the increase in holding time, resulting in the pinning force PZ < the grain growth driving force PG. The abnormal austenite grain growth of four steels appeared at 1000 °C, 1050 °C, 1100 °C and 1150 °C, respectively. The increase in Nb content causes the number of precipitated phases to increase, which increases the temperature and holding time for the appearance of abnormally grown grains.

(3) The calculation of austenite grain growth kinetics shows that the grain growth activation energy Q value increases with the Nb content: 467 kJ/mol for the G20–0.05 Nb steel, 495 kJ/mol for the G20–0.1 Nb steel, 546 kJ/mol for the G20–0.2 Nb steel, and 609 kJ/mol for the G20–0.5 Nb steel. It shows that the increase in Nb content increases the energy required for grain growth and improves grain refinement.

## Figures and Tables

**Table 1:** Chemical composition of SAE4320 steels used in this study (wt.%).

Steel	C	Si	Mn	Cr	Ni	Cu	Nb	Mo
G20–0.05Nb	0.18	0.29	0.61	0.52	1.80	0.11	0.046	0.25
G20–0.1Nb	0.18	0.31	0.61	0.50	1.80	0.11	0.094	0.25
G20–0.2Nb	0.18	0.31	0.57	0.50	1.81	0.11	0.19	0.26
G20–0.5Nb	0.18	0.30	0.57	0.51	1.80	0.12	0.45	0.26

**Table 2:** The growth kinetics formula of austenite grain size and its correlation coefficient.

	Linear Fitting Equation	Related Coefficient
G20–0.05Nb	$D=51,588\times [t\times \exp(-467,358/RT){]}^{0.1975}$	0.98
G20–0.1Nb	$D=22,378\times [t\times \exp(-494,957/RT){]}^{0.1825}$	0.98
G20–0.2Nb	$D=11,189\times [t\times \exp(-545,603/RT){]}^{0.1593}$	0.99
G20–0.5Nb	$D=1801\times [t\times \exp(-609,202/RT){]}^{0.1051}$	0.99

## Data Availability

Data available on request from the authors.
